# Translating purpose and mindset into positive impact through shared vision, compassion, and energy—a comparative study of seven organizations

**DOI:** 10.3389/fpsyg.2024.1251256

**Published:** 2024-01-25

**Authors:** Joseph S. Leah

**Affiliations:** Lutgert College of Business, Florida Gulf Coast University, Fort Myers, FL, United States

**Keywords:** positive impact, greater purpose, shared vision, compassion, relational energy, mindfulness, reflective practices

## Abstract

How do organizations that explicitly state the intention to be “positive impact companies” differ from traditional entrepreneurial companies? How does the quality of relationships in those companies affect the ability to deliver positive impact? This study explores the conditions under which business leaders turn their organizations toward generating prosperity for all stakeholders, achieving positive societal outcomes, improving human wellbeing, and delivering great business results. Seven case study companies are examined based on interviews with three executives from each firm, including the CEO. All seven companies are privately owned small/medium sized businesses from a cross section of industries and diverse geographic bases, ranging from Michigan to the Pacific Northwest, to Singapore, Egypt, and Florida. Four of the companies are considered “positive impact companies” (PICs) based on their organizational affiliations, while the other three are considered traditional entrepreneurial companies (TECs). The overall findings suggest that the core of positive impact leadership resides in a shift in the mindset of leaders toward one of connectedness and purpose, and that these factors influence the quality of relationships in organizations in a positive way whether the company is considered a PIC or a TEC. The results also suggest a close relationship between several core indicators of a fundamental shift in understanding about the role of business in society: shared values of human wellbeing, a common shared vision, an emphasis on collaboration and caring in organizations, and a long-term perspective toward the creation of shared economic prosperity.

## 1 Introduction

The main focus of this study is on the quality of relationships that exist inside organizations, and the role those relationships play in translating an intended greater purpose for the business—and a mindset of connectedness by the leader—into positive impact for the company and society. It continues a stream of research exploring the connection between the mindset and purpose expressed by business leaders and the outcomes their organizations achieve. The quality of relationships is explored in this study primarily through the lens of theories related to relational climate (Boyatzis and Rochford, 2020) while the societal impact of business is explored through the lens of theories related to flourishing enterprise (Laszlo et al., 2014). The study aims to provide evidence for further scholarly exploration of the underpinnings of relational climate theory and flourishing enterprise theory (and the intersection between them) while offering points of consideration for practitioners seeking to deliver positive societal impact through their business organizations.

Many studies in the related literature are compartmentalized, with emphasis targeted at the level of the individual, team, or organization, or aimed at broad meta-analyses offering evidence for societal trends. The motivation for this study is to explore the thread that links these levels, from the mindset of individual leaders through the quality of relationships in the organization to the impact on external stakeholders. An additional motivation is to explore the differences between organizations that actively seek positive impact as part of their reason for being, versus the impact from those focused on conducting successful business in a more traditional sense.

The exploration of the role of business as part of the broader societal and environmental context is timely and relevant. A range of organizations and initiatives suggest that a consensus may be emerging about a shift in direction for the future of business – from the Earth Charter (The Earth Charter Commission, 1992) to the sustainable development goals of the United Nations (United Nations General Assembly, 2015) to the COP28 consensus (The Economist, 2023) and many others. The 2019 Business Roundtable statement on the purpose of the corporation expressed a stakeholder perspective with a focus on long-term value (Business Roundtable, 2019), while the emergence of B Corporations, and the growing influence of environmental, social, governance investment approaches, each contribute to a shift in the understanding of the role of business in society.

This study focuses on companies and leaders who are known to be conducting their activities with an aim toward becoming “positive impact companies” (PICs)—committed to generating prosperity for all stakeholders, achieving positive societal outcomes, and improving human wellbeing—and to explore the conditions under which positive social and environmental outcomes emerge in tandem with positive economic performance. In comparison will be “traditional entrepreneurial companies” (TECs) known for conventional business success.

The research approach incorporates a comparative case study analysis based on four companies identified as PICs, and a comparison group of three companies identified as having success as TECs, but without any such explicit aim toward a greater purpose. Interviews were conducted with three executives in each company, including the CEO. This allowed for an in-depth analysis of the dynamic between the individuals in each organization and the opportunity to address any self-reporting limitations by engaging with multiple respondents within an organization.

The objective is to explore several perspectives from the same organization, allowing for the organization to be addressed as a unit of analysis, as well as allowing for an evaluation of the quality of relationships in the organization. The observations are based on the perceptions of individual members of those organizations related to organizational performance, derived through qualitative interviews. Two overriding questions animate the exploration of the seven cases: (1) How does the quality of relationships in an organization affect the ability to translate the leader’s mindset and purpose into positive impact for stakeholders? and (2) How do organizations identified as PICs differ from traditional entrepreneurial companies?

### 1.1 Theoretical framing

A renewed interest in the study of human flourishing highlights the centrality of connectedness both in terms of personal wellbeing as well as in terms organizational impact. More broadly, there is a growing understanding of the link between a mindset of connectedness within individuals (especially leaders), the impact on the quality of relationships within their organizations, and the resulting global impact on positive societal outcomes. The concept of oneness and interconnectedness permeates virtually all spiritual traditions, while at the same time the latest developments of modern science and a rediscovery of ancient philosophies are driving many toward a new understanding of human connectedness (Laszlo et al., 2014).

The core of the theoretical construct of this study begins by exploring the mindset of the leaders of each of the seven case companies, the extent to which they adopt reflective practices that may inform their sense of connectedness, and their expression of a possible greater purpose for their organizations. It then explores how those core elements influence the quality of relationships in those organizations, ultimately leading to positive impact outcomes.

**Figure 1:**
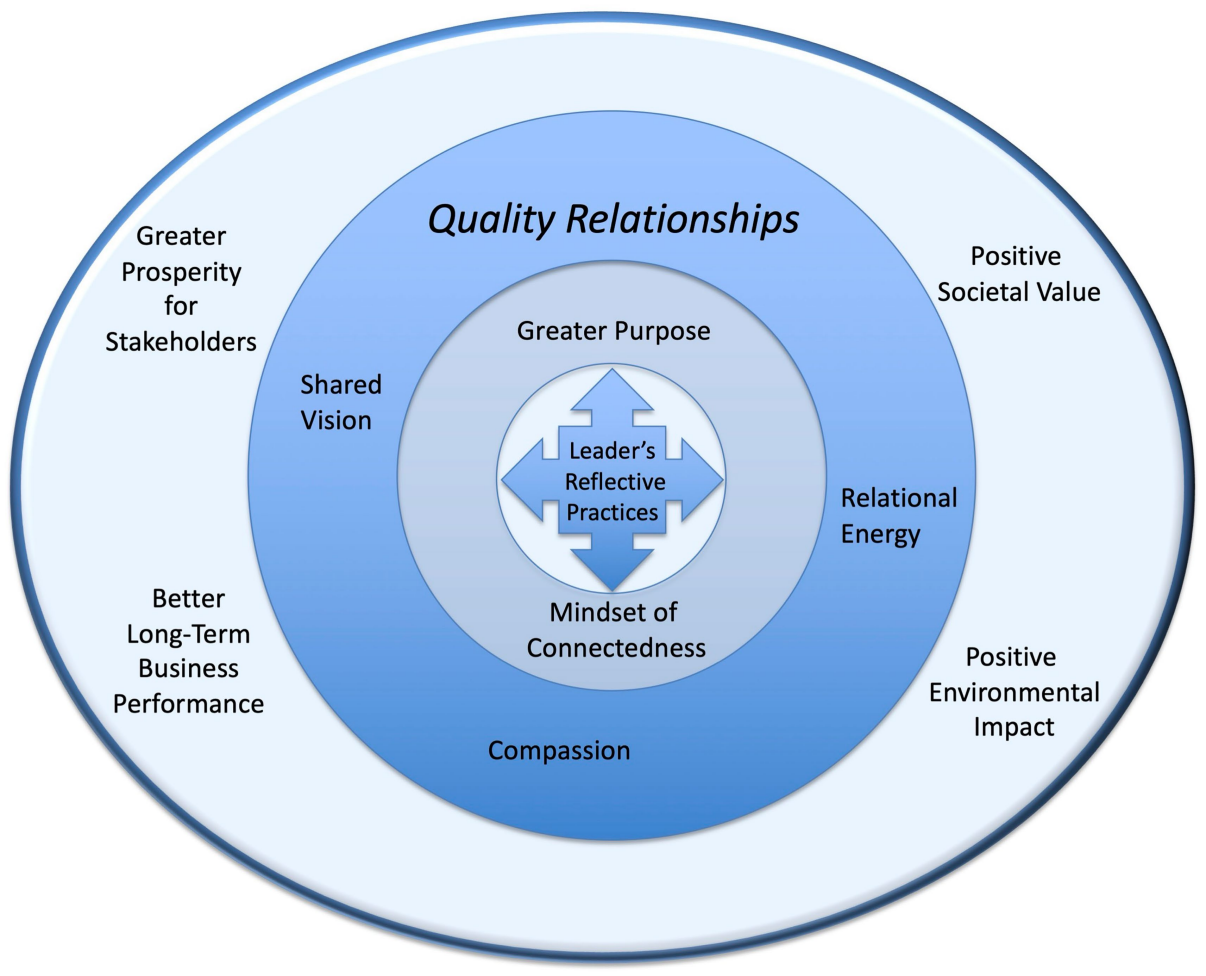
Quality relationships in organizations translate leader mindset and purpose into positive outcomes.

The overall theoretical framing is visually expressed in Figure 1. Within this framework of understanding, the area of focus shifts as attention moves from the personal to the organizational to the societal. At its core are personal practices by leaders, resulting in an emerging sense of connectedness and the expression of a higher purpose for the organization. Broadening the field of view takes the focus to the level of the organization, and the relationships that develop within the organization and the extent to which purpose and sense of interconnection are shared. At its widest angle, the focus shifts to the business’ impact on various stakeholders within its business system and in society.

Each layer within this model draws on a deep field of literature. The review below explores some key components of that literature, starting at the center of the lens with an emphasis on individual leaders, then moving to the level of relationships and shared interactions, and finally discussing literature related to the positive impact of business in society. The identification of the main theoretical themes within this framework were derived from the literature across each of the levels explored in Figure 1. For example, one recent study validated the correlation between the main theoretical constructs through a quantitative study (Leah and Laszlo, 2022), while another explored the qualitative nature of reflective practices and a mindset of connectedness (Tsao and Laszlo, 2019). Boyatzis and Rochford (2020) have conducted extensive work in the study of the quality of relationships, while the correlation of positive relationships, mindset and purpose with increased organizational outcomes builds on findings from Whelan et al. (2021), Friede et al. (2015), Eccles et al. (2014), and others.

#### 1.1.1 Leader mindset of connectedness and reflective practices

A mindset of connectedness is often associated with regular practices of reflection. Tsao and Laszlo (2019) report that out of 34 business leaders rated as ‘high’ on the level of consciousness using their analytical criteria, 32 expressed regular usage of practices of connectedness in their leadership approach. The working definition of reflective practices includes both “spiritual” types of activities such as prayer or meditation, as well as “non-spiritual” activities such as journal-writing, exercising, walking in nature, gardening, making art or similar non-spiritual activities (Laszlo et al., 2014). The underlying principle of a reflective practice is to identify a regular activity that allows an individual or group to stop and think, to contemplate, to develop a sense of connection to what they are doing and to the world around them.

The value of reflective practices is to quiet the mind and allow us to perceive signals that otherwise would have been missed. They can serve to help overcome the “blindspots” that emerge in a leader’s thinking that inhibit the full attention required for dealing with a rapidly changing environment (Scharmer, 2000). Senge et al. (2015) describe reflection as one of the core competencies of what they call system leaders, enhancing the ability of leaders to engage in more meaningful conversations, challenging taken-for-granted assumptions, and overcoming limiting mental models. They further suggest that shared reflection can enable groups of individuals to more deeply understand each other’s points of view at an emotional as well as cognitive level. Scharmer (2009, p. 18) describes the process as one of “co-presencing: go to the place of individual and collective stillness, open up to the deeper source of knowing, and connect to the future that wants to emerge through you”.

Boyatzis and McKee (2005) emphasize mindfulness as a source of renewal and define it as “living in a state of full and conscious awareness of one’s whole self, other people, and the context in which we live and work.” Additionally, they suggest that the experience of compassion with people around us is deeply renewing, and is defined as a “combination of deep understanding, concern, and a willingness to act on that concern for the benefit of oneself and others”.

Another perspective on the impact of reflective practices is to view them through the lens of intentional change theory (Boyatzis and Akrivou, 2006) and the perspective of renewal practices. Boyatzis and Akrivou (2006) propose that the ‘ideal self ‘is a powerful driver in the framework of intentional change, and that three components converge toward the expression of one’s ideal self: an imagery of a desired future, the emotional fuel of hope, and the enduring set of personal characteristics of a person’s core identity. One consideration is whether reflective practices allow people to tap into all three of these components, and to connect with their true core identity by quieting the noise and pressures of life, and the distortion of what Boyatzis calls the ‘ought self’. Seen through this perspective, reflective practices may serve as a pathway toward uncovering an ideal self that is aligned with positive impact leadership.

Intentional change theory informs thinking not only at the personal level, but also at the dyad, team, organizational and societal levels. It offers an effective framework for considering the evolution of ideal self from the perspective of individual leaders, companies, industry sectors, and the overall institution of business.

In terms of personal wellbeing, Jon Kabat-Zinn has written extensively on mindfulness practice as it relates to health, human interactions, and positive outcomes. His technique of mindfulness-based stress reduction (MBSR) has attained widespread application in a range of business, government and non-government organizations, and has been demonstrated to provide measurable positive health outcomes (Kabat-Zinn, 1994). As one of an extensive range of studies related to MBSR, Brewer et al. (2011) explored default mode network activity and connectivity related to meditation experience, and found decreased mind-wandering in experienced meditators.

A leader mindset of connectedness (developed through reflective practices), combined with an expression of greater purpose for the organization has been found to have a meaningful impact on the quality of relationships in an organization and measures of relational climate (Leah and Laszlo, 2022).

#### 1.1.2 Mission and purpose of the organization

A range of studies have explored the impact of purpose on organizational culture and performance. A recent eight-year study of high-growth companies found that, among four drivers of growth examined by the authors, purpose boosted growth the highest. Their report suggests that a purpose-driven strategy helps companies address business challenges such as slowing growth and declining profits, while also providing three specific benefits described as: “more-unified organizations, more-motivated stakeholders, and a broader positive impact on society” (Malnight et al., 2019, p. 79).

The presence of a “greater purpose” as part of the organization’s reason for being has been identified as a component of an environment that positively influences intrinsic motivation of employees (Ryan and Deci, 2000; Gagné and Deci, 2005). Research on the impact of mission statements suggests that, when expressed in terms of a social or environmental issue rather than a stakeholder group, there is a significant impact on behavior in those firms regarding the issue (Bartkus and Glassman, 2007). The idea of purpose and meaning at work is a central aspect of the study of workplace spirituality. In their scale developed to measure meaning at work, Ashmos and Duchon (2000, p. 143) incorporate several items geared toward purpose. Two items in the scale were: “The work I do is connected to what I think is important in life” (item 8) and “I see a connection between my work and the larger social good of my community” (item 10).

The ability of leaders to express an appropriate vision and to create an environment where greater purpose and flourishing can emerge has been explored as a core element of leadership. The field of Appreciative Inquiry takes as one of its foundations the power of “dreaming” about an ideal future (Cooperrider and Srivastva, 1987). The creation of an organizational framework that allows individuals to flourish has been described as representing the next step in the continued evolution of organizational development toward a better world, driven by a noble mission that has a powerful impact on the long-term engagement of employees and overall firm performance (Laloux, 2014).

While research on the entrepreneurial orientation of social entrepreneurs compared to traditional commercial entrepreneurs indicates that the primary drivers of entrepreneurial success are essentially the same in both contexts (Lumpkin et al., 2011), further research focused on entrepreneurs suggests that the perspective on spirituality and higher purpose of the individual entrepreneur is an important differentiating factor in understanding their behavior and motivation (Kauanui et al., 2008).

When leaders are perceived to operate in a way that suggests a higher purpose, for example with a clear sense of corporate social responsibility, the impact on people inside the organization has been found to lead to positive outcomes. Glavas and Kelley (2014) report the results of a study of 827 employees in eighteen organizations, showing that employee perceptions of corporate social responsibility (CSR) are positively related to organizational commitment, with the relationship being partially mediated by work meaningfulness and perceived organizational support. In a similar vein, Lavine (2012) has reported that a strong relationship exists between corporate social performance and work meaningfulness reported by employees. Further, in a study of 322 business leaders, Leah and Laszlo (2022) reported a strong direct relationship between greater purpose and the achievement of positive societal outcomes (standardized estimate of 0.331, *p* = 0.001) and an even stronger positive correlation with relational climate (standardized estimate of 0.351, *p* < 0.001).

#### 1.1.3 Quality relationships in the organization

The core of this study’s exploration of quality of relationships as a critical mediator between purposeful intention and positive impact is based on the construct of relational climate built on the interrelationship between three sub-constructs: shared vision, compassion, and relational energy (Boyatzis and Rochford, 2020). Shared vision refers to a mental image of a positive future state that forms the basis for common action for members of an organization. Compassion relates to going beyond empathy to noticing another person’s need and taking action to improve their wellbeing. Relational energy describes how relationships in an organization increase positive energy and embrace a feeling of aliveness and readiness to act.

The impact of positive relational climate, compassion and relational energy builds on extensive research into the impact of positive and negative emotional attractors in organizations (Boyatzis et al., 2015). Previous studies built on advances in neuroscience to measure and demonstrate the impact of resonant leadership and positive relationships in the brains of followers, indicating that the impact is medically and scientifically measurable (Boyatzis, 2012). Research on the neurological impact of empathy indicates that “consistent evidence shows that sharing the emotions of others is associated with activation in neural structures that are also active during the first-hand experience of that emotion” (Singer and Lamm, 2009). Further research has explored neurodevelopmental changes in the underlying circuits of the brain relating to empathy and sympathy (Decety and Michalska, 2010) and the affective neuroscience and its mechanisms (Decety, 2011).

The quality of relationships remains an important factor in the face of increasing levels of remote work and organizational dispersion. Lorca and Belli (2023, p. 10) have explored the impact of leadership behaviors on the relational climate in organizations with high levels of hybrid work. Their findings suggest that maintaining a strong climate in such a context requires leaders to walk a tightrope—which they describe as funambulist leadership—between creating an environment of feeling valued and the responsibility to achieve organizational outcomes. A further field study by Lopez Carrasco and Belli (2023) explored the emotional dimension of scientific research groups, reinforcing the finding of a need for balance in managing the relational climate between affection, warmth and spontaneity within a group and the individual strategies of the leader.

In their book “Resonant Leadership,” Boyatzis and McKee (2005) explore in depth the centrality of emotional connection to effective leadership. They provide a range of arguments on the impact of a sense of connectedness on others inside an organization. One aspect they explore relates to emotional contagion – both positive and negative – and the idea that we have an innate ability to pick up nearly imperceptible clues from one another as to our emotional state and intentions. Hazy and Boyatzis (2015) discuss emotional contagion in organizations, and argue that the drive for human cooperation may have preceded, in an evolutionary biology sense, the cognitive processing necessary to make complex rational decisions. They go on to explore the relationship of positive emotional attractors (PEA) and negative emotional attractors (NEA) with emotional contagion and argue that either of those emotional states could be contagious under certain conditions.

The role of the PEA/NEA also forms the core of a study by Boyatzis, Rochford, and Taylor of the impact of a leader’s emotional state on shared vision and effective leadership and relationships. Boyatzis et al. (2015) argue that for a leader, a personal vision based on an “ideal self” is a required starting point toward leading successful desired change. They further argue that the leaders, and others in the organization in the case of shared vision, must be in a PEA mindset while creating the vision based on ideal self. While negative emotional attractors are necessary to spur action, sustained desired change requires significantly more time in PEA than NEA.

Taken together, the studies from Boyatzis and others suggest that the mechanism by which a leader’s mindset, sense of connectedness and purpose are infused within the relational climate of an organization can be understood through the concept of emotional contagion and positive/negative emotional attractors. The quality of relationships is interconnected with the mindset of leaders and the purpose of the organization.

#### 1.1.4 Perceived business performance and positive impact outcomes

Positive business outcomes are defined as increasing economic prosperity while contributing to a healthy environment and improving human wellbeing (in contrast to traditional sustainability approaches that are primarily focused on doing less harm). Sisodia et al. (2014) have conducted an ongoing stream of research aimed at exploring what they call “firms of endearment, “which in many ways display positive business outcomes. A core finding of their analysis is that positive impact approaches to business tend to achieve greater long-term performance.

Eccles et al. (2014) have compared the financial performance over eighteen years of companies identified as “high sustainability” firms to the financial performance of “low sustainability” firms. High sustainability firms were identified as those who integrated social and environmental policies as part of their business model. Their analysis found that High Sustainability firms achieved better business and stock market performance than Low Sustainability firms.

Laszlo and Zhexembayeva (2011) have demonstrated the strong business case for integrating positive environmental and social outcomes as a source of long-term competitive advantage and economic prosperity. Laloux and others have explored the evolution of the organizational context (Laloux, 2014) as part of the evolution of business toward shared prosperity and positive social and environmental outcomes.

Finally, two recent meta-studies provide additional evidence to the correlation between positive impact outcomes and business performance. Friede et al. (2015) aggregated all related academic reviews from 1970 to 2014 and found that 90% of studies reported a positive or neutral correlation between corporate financial performance and positive environmental, social and governance measures. Whelan et al. (2021) reported similarly positive correlations in a meta-study of 1,000 studies published between 2015 and 2020.

#### 1.1.5 Summary of theoretical framing

Much of the literature exploring the interrelationship between purpose, leader mindset, relational climate and organizational outcomes is based on measuring perceptions of individuals within organizations. While these are valuable points of exploration, a gap emerges in the understanding of relational climate when measuring only the perceptions of a single individual in an organization.

By evaluating the perceptions of several individuals in each case study organization, it is possible explore the layer of “quality relationships” depicted in Figure 1 more fully. This is the leverage point at which the mindset of leaders, and the intention they may express for their organizations, is translated into outcomes for multiple stakeholders.

## 2 Methods

The primary research method of this study is the development of a comparative case study assessment of seven organizations based on analysis of themes drawn from the literature. The study has been designed to explore both the perspectives of individual leaders as well as the level to which those perspectives are shared in the case organizations. It achieves this by measuring perceptions of several individuals from the same organization through separate, protocol-led interviews followed by coding and analysis to identify points of convergence. This approach allows for identifying perspectives that can more reasonably serve as representative of the organization, rather than the perception of a single individual. The interview protocol is designed to address each layer of the theoretical framework depicted in Figure 1.

### 2.1 Research design

The selection of seven companies to serve as case studies provides an opportunity for comparison of the perceived performance of each company from multiple people in the organization and allows for better understanding of the relationship between leader actions and social and environmental outcomes and business performance.

The intent of this case study analysis is twofold: to provide validation of comments from leaders of each company, therein providing increased reliability on assessment of the organization relative to each of the constructs being explored; and to offer a comparative analysis between companies pre-identified as PICs relative to those pre-identified as TECs. The outcome of the case study analyses offers a clearer picture of the conditions under which the various components of the theoretical model converge.

The interviews were conducted in a conversational format at the company offices, an offsite location, or virtually by Zoom. The interview length ranged from 40 to 60 min each. Research was conducted in accordance with the Belmont Report (United States National Commission for the Protection of Human Subjects of Biomedical and Behavioral Research, 1978), with all plans for research submitted in advance to the Institutional Review Board of Case Western Reserve University. Each company provided a letter of cooperation to conduct research inside their organizations.

The subsequent analysis was conducted following a coding process within each *a priori* thematic area, and a comparative evaluation of individual inputs from each case organization. Initially eight thematic areas were evaluated and compared for convergence within each of the seven case companies. This was expanded to nine thematic areas following the initial analysis process, to account for the emergence of alternative expressions of mission/purpose and core values. The nine areas of thematic evaluation were: leader’s reflective practices, mindset of connectedness, mission/purpose, core values, shared vision, compassion, relational energy, positive economic outcomes, and positive social/environmental outcomes. The comparative evaluation of the seven cases is summarized in Table 1.

**Table 1:** Summary of presence/absence of themes by case.

Case study	Pre- ID	Explicit GP	Core values	Shared vision	Relational energy	Compassion	MC	Practices	Econ perf	Soc/Env Perf	Total
Case #1 – Heavy Mfg	PIC	0	1	1	1	1	1	1	0	0	6
Case #2 – IT/Design	PIC	1	0	1	1	0	1	1	1	1	7
Case #3 – Mfg B2C/B2B	PIC	0	1	1	1	1	1	1	1	1	8
Case #4 – Multi-biz	PIC	0	1	1	1	1	1	1	1	1	8
Case #5 – Roofing/Constr.	TEC	0	1	1	1	1	0	0	0	0	4
Case #6 – Hardware	TEC	0	1	1	1	1	0	0	0	0	4
Case #7 – Mfg – B2C	TEC	0	0	1	0	0	0	1	1	1	4

#### 2.1.1 Company selection approach

The initial company selection was focused primarily on PICs. Four companies were recruited as having an explicit PIC-oriented purpose, and three control companies of similar size/industry profile were recruited from a group of successful regional businesses (using traditional business metrics with no explicit PIC-purpose). Small/medium-sized companies were emphasized in case recruitment.

The reasons for focusing on small/medium-sized companies include: large multinational corporations have been more widely studied in terms of social and environmental leadership, while a gap exists in the understanding of small/medium-sized enterprises; at the same time, a review of the business and entrepreneurship press suggest that many of the innovations toward social and environmental value are emerging from small/medium-sized enterprises; the opportunity to access the ownership and senior executive levels of small/medium sized enterprises is greater than with large multinationals, and will allow a deeper exploration of the direct interaction between the leader’s vision and purpose for the organization and its expression and adoption within the organization. The companies were identified and recruited from a population of companies known to pursue positive social and environmental outcomes based on their participation in any of a number of organizations, including participation in the Global Forum series hosted by the Fowler Center for Business as an Agent of World Benefit, the list of B-Corporations, Conscious Capitalism, and other similar sources.

Once the initial process for selection of PIC companies was established, the three TEC companies were recruited to roughly reflect similar company size, industry, and other demographic measures. These companies were identified for traditional entrepreneurial business success, with no known objective toward social or environmental outcomes as part of their business mission.

All of the case study companies are small/medium sized enterprises (SMEs), and all are privately held. The four PICs were represented by a heavy manufacturing and industrial service company, a software engineering and design company, an international manufacturing company making products for both consumer and business-to-business applications, and a multi-business company involved in heavy industry, construction, real estate and direct to consumer services. The three TECs were represented by a large regional roofing construction company, a manufacturing company of consumer products, and a large regional hardware company.

#### 2.1.2 Interview subject selection approach

Three executives were interviewed from each of the selected companies, with the interview subjects holding approximately the same level and role in each organization. I attempted to speak with the following three roles within each company: an owner/senior executive who would be a leader in setting the purpose and vision for the enterprise; a senior executive in an administrative/HR role, who would be able to provide a perspective on the organizational climate, and a mid-to-senior manager in an operational role who could provide a line perspective on the objectives, climate and activities of the company.

#### 2.1.3 Interview protocol approach

The interview protocol for the case studies is designed to further explore the constructs and conceptual framing of the overall theoretical model. This approach is designed to allow a deeper exploration of the core concepts of the overall research, as well as to facilitate triangulation and explanatory findings. The interview protocol is available in Table 2.

**Table 2:** Interview protocol.

1	How would you describe the mission and purpose of your organization?
2	Do you believe there is a shared vision, sense of purpose, in your organization? Among whom is it shared? If there is a shared vision, could you please share with me a couple of examples of how everyone in the organization understands and works toward the organization’s stated vision/mission?
3	Do you believe there is a compassion or caring for each other in your organization? Among whom is it shared? If there is a shared compassion, could you please share with me a couple of examples of how people in the organization interact with each other in emotionally challenging circumstances?
4	Do you believe there is a shared energy about your relationships in your organization? Among whom is it shared? If there is a shared relational energy, how would you describe the energy level inside your organization?
5	If I were to ask: “I feel most deeply connected to ______” – what would you put in the blank?
6	When you feel most connected, what does it feel like, or what are you experiencing? What kinds of practices, routines or activities do you incorporate into your leadership activities (either yourself or with your team) that increase your sense of connectedness to self, others, and the world (like prayer, meditation, keeping a journal, going for a walk in nature, etc.)?
7	How do you perceive your organization’s performance on the following measures of business performance, relative to your major competitors: Performance in revenue growth; Performance in profitability; Performance in acquisition of new customers
8	How do you perceive your organization’s performance on the following measures of social/environmental performance, relative to your major competitors: Design of products or services that provide solutions to environmental or social problems; Partnering with suppliers that use socially and environmentally friendly practices; Acting as a responsible corporate citizen in the local community; Creating innovations aimed at increasing social or environmental value

The protocol is designed to approach the discussion first relative to perceptions of the company, then about the leader’s personal perspective and practices, and finally about perceptions regarding the company’s performance. Each of the themes derived from the literature and identified as part of the model in Figure 1 is addressed in the interview protocol, although not in a linear order. The intent of opening with a broad question about the mission and purpose of the organization is to elicit the interview subject’s top of mind response, before engaging in the deeper discussion about the organization.

The protocol and discussion style are designed to avoid as much as possible the entry of biases, such as social desirability, that would influence the responses. Interviews were conducted separately and independently with each interview subject.

### 2.2 Analysis process

The structure of the case analysis was informed by an adapted approach to thematic analysis based on Boyatzis’ “Transforming Qualitative Data: Thematic Analysis and Code Development” (Boyatzis, 1998), as well as Yin’s “Case Study Research: Design and Methods” (Yin, 2014).

The thematic analysis of this study was based on themes derived from the overall theoretical framing of the study. The interview protocol was designed to explore these themes while still maintaining a conversational approach to the qualitative interviews. As the analysis of the interview transcripts progressed, however, one of the *a priori* themes was being expressed in two distinct ways by the interview subjects. As a result, that theme was split to capture the distinction that emerged between the expression of an explicit greater purpose as part of the business mission, and the expression of a set of positive core values as the guiding direction for the business. As will be shown in the review of findings, only one company (a PIC) showed convergence across the interview subjects on the expression of greater purpose. The other three PICs showed convergence around a set of positive core values. None of the TECs showed convergence on a greater purpose, but two converged on positive core values.

While the unit of observation in this study is based on individual perceptions of the company, the unit of analysis is intended to be the organization. As a result, the process of analysis includes several steps in an effort to systematize the interpretation of the qualitative data from the interview transcripts, and to provide a reasonable assessment whether a given state – relative to each theme – is either present or absent in each company (Boyatzis, 1998). The reliance on self-reported perceptions of company performance creates the potential for single response bias as a limitation. While various studies have validated the reliability of self-reported economic performance in research (Liozu and Hinterhuber, 2013), the potential for response bias is mitigated by analyzing the convergence of perceptions across several interview subjects.

The process seeks to determine the presence/absence based on whether a convergence exists across the three interview subjects, and then whether that convergence is positive (presence) or negative (absence). The process steps include:

1. Each transcript was coded at a high-level application of the pre-existing themes of the study. Coded excerpts were extracted as evidence of the respondent’s comments related to the constructs.
2. The coded excerpts were scored on a scale of 1 to 5 based on my assessment, as the investigator, of the alignment of the excerpt with the concept being measured. In order to ensure a reasonable level of consistency and reliability in my assessment, I applied both a holistic evaluation as well as an assessment informed by the themes of the study (similar to a rubric that a professor might use in grading papers)
3. The extracted excerpts were aligned side-by-side in three columns (one for each of the three interviewees) and were arranged in rows according to the theoretical themes
4. The three excerpts were compared relative to the corresponding theoretical theme to determine a face assessment of convergence, and the three numerical scores were compared
5. If the three separately applied scores combined were within one standard deviation of each other (indicating adjacency across the three scores), and the visual interpretation confirms, then a state of convergence was applied to that company on that measure
6. To determine presence or absence, if convergence exists, and the mean of the scores of the three transcripts was at 4 or greater, then a state of presence was applied for that company on that theme (and given a Boolean score of 1)
7. If convergence exists, but the mean of the scores was less than 4, then a state of absence was applied (a Boolean score of 0)
8. If no convergence exists (either through a standard deviation of the scores greater than 1, or a lack of visual confirmation), then a state of absence likewise was applied (a Boolean score of 0)

The presence/absence scores were then used as the basis for the findings described in the next section. A description of each of the seven case companies is provided Table 3. The complete comparative convergence table is shown in Table 1.

**Table 3:** Overview of the seven case companies.

Case Company	Industry	Company description	Location	Designation	Interviews conducted
Case #1 – PIC	Heavy Manufacturing Company	Large privately held shipbuilding and industrial service company based in Pacific Northwest. Transforming culture toward one based on a set of clear core values described as truth, responsibility, evolution, and love.	USA, Pacific Northwest	Selected as PIC due to strong affiliation with Fowler Center for Business as an Agent of World Benefit.	The CEO, the VP of HR, and an Operational Site Manager
Case #2 – PIC	IT/Engineering and Design Company	Privately held IT, software engineering and design company based in Michigan with highly unique culture.	USA, Michigan	Selected as PIC due to affiliation with Fowler Center for Business as an Agent of World Benefit at Case Western Reserve University, and for its publicly known innovative approach to creating positive impact.	The CEO and two separate Project Leaders
Case #3 – PIC	Manufacturing Company – B2C and B2B products	Privately held manufacturing company of packaging materials for direct-to-consumer products as well as business-to-business products. Headquarters in Cairo, Egypt. Approximately 400 employees.	Egypt	Selected as PIC due to affiliation with Fowler Center for Business as an Agent of World Benefit at Case Western Reserve University, and for its active system wide application of appreciative inquiry approaches in the company.	The CEO, the VP of Operations, and the VP of Engineering
Case #4 – PIC	Global Shipping and Multi-Business Company	Privately held 4^th^ generation family business in multiple sectors including industrial shipping, real estate, investments, and direct to consumer services.	Singapore	Selected as PIC due to close affiliation with Fowler Center for Business as an Agent of World Benefit at Case Western Reserve University.	The CEO, a Divisional VP, and the Head of HR
Case #5 – TEC	Roofing and Construction Company	A second-generation family-owned business. One of the largest regional roofing and construction companies in Florida.	USA, Florida	Selected as a TEC for entrepreneurial and traditional business success in a highly competitive environment.	The CEO, the VP of Operations, and the VP of Sales
Case #6 – TEC	Hardware and Home Improvement Company	A multi-generation family-owned business. Competing in a highly challenging environment against big home improvement firms on one side (Home Depot, Lowes), specialized competitors on the other side, and Amazon across sectors.	USA, Florida	Selected as a TEC for entrepreneurial and traditional business success.	The CEO, the VP of Operations, and the General Manager of one of the retail stores
Case #7 – TEC	Consumer Products Company	A privately held entrepreneurial startup with rapid growth in the manufacturing of healthy lifestyle products.	USA, Florida	Selected as a TEC for entrepreneurial and traditional business success.	The CEO, the VP of Operations, and the VP of Commercial Sales

As an illustrative example of responses that were evaluated as showing convergence with presence of the theme, the following excerpts are from Case #2 in response to the first protocol question regarding the mission and purpose of the organization:

Case #2:

Response from the CEO: *“My Co-Founder and I and we have been running this for 16 years. We decided a couple of big things, big ideas. We wanted to end human suffering in the world as it relates to technology.”*

Response from Executive 1: *“So we have a formal answer to that and by formal, I mean a shared mission statement where that is to end human suffering in the world as it pertains to technology and both the use of technology and the creation of technology,”*

Response from Executive 2: *“End human suffering in the world as it pertains to technology.”*

In this case, the CEO and both executives independently offered a clearly stated, consistent response suggesting a strong sense of the organization’s purpose.

As an illustration of responses showing no convergence, the following excerpts are from Cases #1 and #3 in response to the same question regarding mission and purpose:

Case #1:

Response from CEO: *“I see the organization’s purpose around this being the profitability, make money and make a difference and if it’s not doing both it is not doing anything.”*

Response from Executive 1: *“We do not have a formal mission, we do not have a, you know, a formal purpose….”*

Response from Executive 2: *“We did some work around values and so, in my own mind it starts with values, we determine our values, our truth, responsibility…*”

Case #3:

Response from the CEO: *“Maybe over 10 years ago we had the purpose and the mission of we deliver quality and then it shifted a little bit. And then we said we care about our customers. And then it shifted a few years later by just putting our customers between these two… And then just recently like two years ago, we shifted it again.”*

Response from Executive 1: *“I think that <company> is somehow different from the other two companies I’ve worked for… <company> is focusing on the person itself and how it’s important to the company and to the community… just be positive, focus on the work you are doing and avoid any negative clients or any negative remarks around yourself.”*

Response from Executive 2: *“<Company> mission is to be the best company for the people who’s working in <company> for the <company> customers and <company> suppliers.”*

Although all three of these companies were PICs, the responses from Cases #1 and #3 suggest that the mission and purpose of the organization is either not well established or has not been effectively communicated. In Cases #1 and #3, the analysis determined that no convergence existed in these organizations for mission and purpose.

Across each of the seven case companies, the responses from the CEO and two additional executives were evaluated following the eight evaluation process steps outlined above.

## 3 Results

The traditional entrepreneurial companies that I interviewed were anything but traditional upon closer examination. They all demonstrated at some level the core components of the PICs. It is clear through this analysis that there is no black and white, PIC vs. TEC dichotomy in evidence. Rather, there is a spectrum of evolution along multiple dimensions of performance and positive impact.

**Figure 2:**
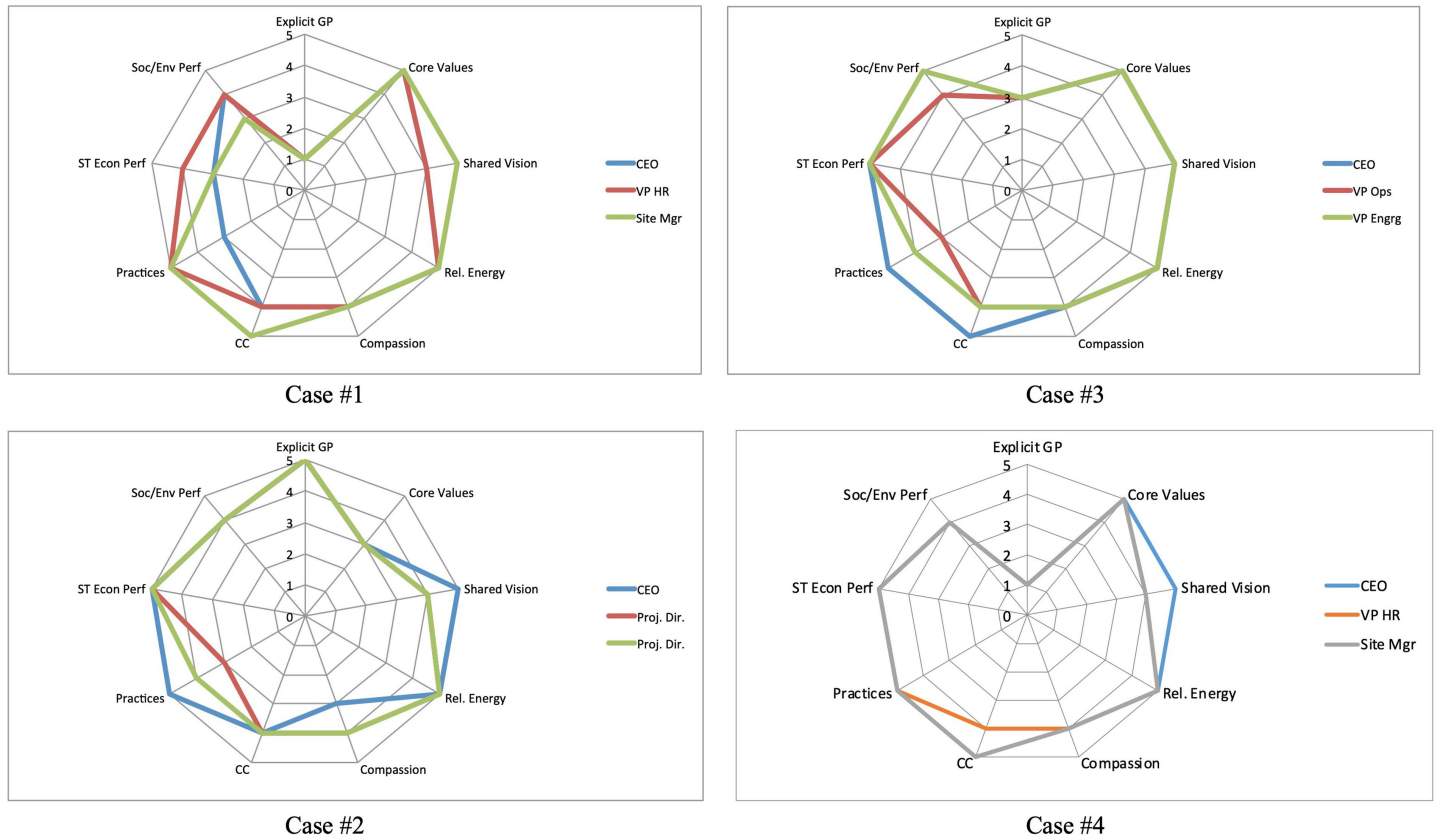
PIC case companies – visual representation of the level convergence across nine thematic areas.

**Figure 3:**
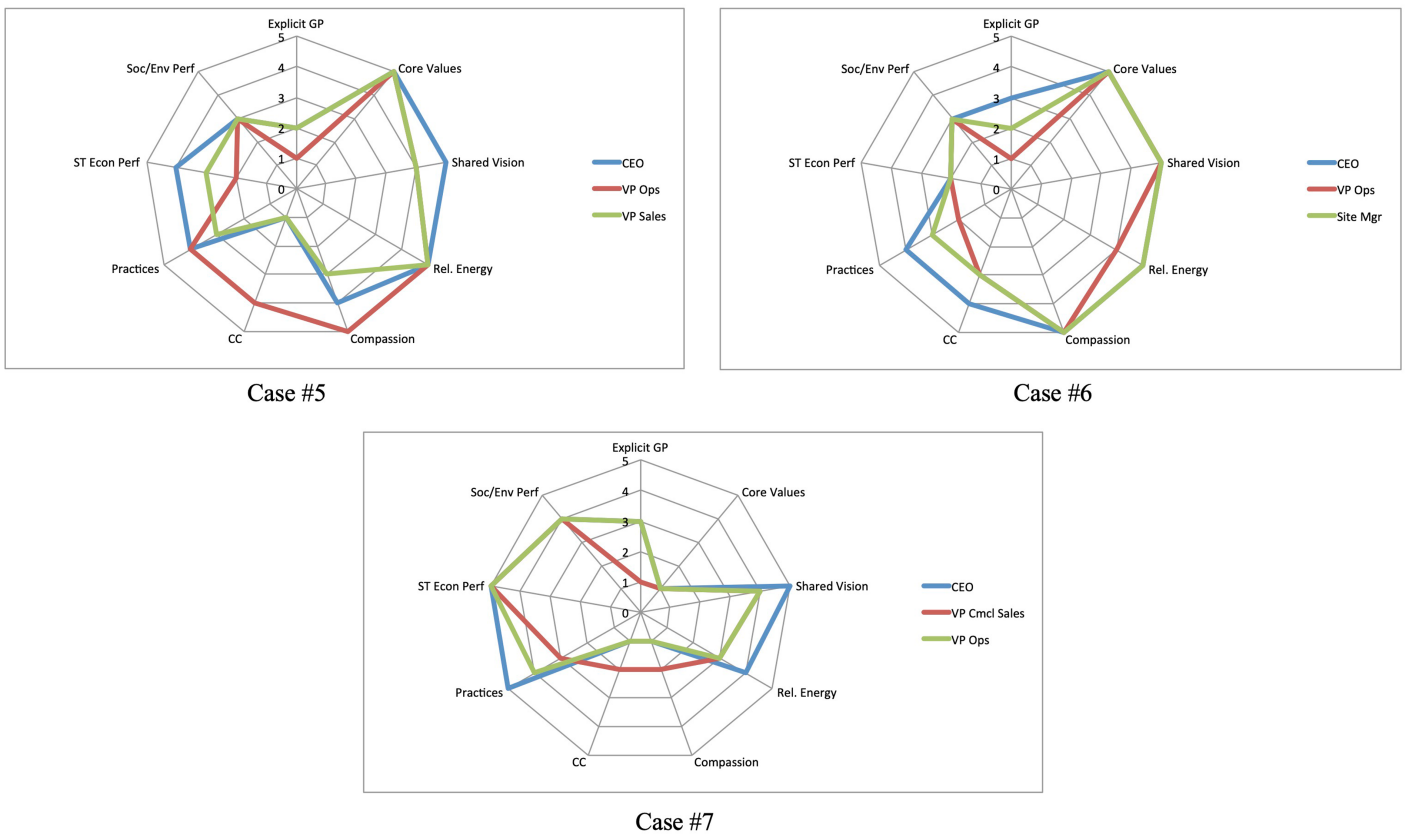
TEC case companies – visual representation of the level of convergence across nine thematic areas.

The analysis of the convergence results from Table 1 was plotted onto radar charts for visualization. The convergence charts for the PICs are shown in Figure 2, and those for the TECs are shown in Figure 3.

### 3.1 Finding #1—shared vision is the only theme that was convergent in all seven cases

Only one theme out of the nine evaluated showed convergence across all seven companies, regardless of whether PIC or TEC. That theme was shared vision. It is a core component of relational climate, and as such, is a key factor in creating quality relationships. It represents a central component of the original goal of this study, which is to explore how the quality of relationships in an organization affects the ability to deliver positive impact outcomes. This finding aligns with the assertions of a range of studies, beyond the scope of this paper, suggesting that organizational culture is a powerful force when there is clear alignment with that culture by people in the organization.

Shared vision is distinct from mission/purpose. It is a measure of how much alignment exists behind a given mission/purpose in an organization, whatever it may be. Across the seven cases in this study, the expression of mission or purpose was more commonly presented in terms of core values (see Finding #3). However, whether expressed as higher purpose or as a set of core values, alignment was consistent across all seven cases. This finding illustrates the critical role of quality relationships in translating the mission/purpose or core values into positive impact outcomes. The following excerpts illustrate the varying ways this concept is expressed among the interview subjects:

Case #2:

Response from CEO: *“It would be very difficult here for a number of different reasons for [the culture] not to be widely shared among the team… So, when I talk about our culture, what I’d say is that what we have is an intentional culture.”*

Response from Executive 1: *“I believe in my gut, in my heart, as harder things get, sometimes, we really do all share a particular vision of what we are trying to strive for, how we want to treat each other, what we want our culture to be, how we want our system and our structure to facilitate and help that kind of culture grow. I know we all share that very deeply and along with that is, you get very comfortable in some of that, that culture.”*

Response from Executive 2: *“<Company> as an organization strives towards being deliberate in creating its culture and creating a shared vision more than any other sort of organization or workplace that I’ve been at.”*

Case #3:

Response from the CEO: *“Is it a shared purpose or a vision, I’m not sure but it’s a shared in the sense of destiny, sense of being together, sense that we are all in this and we’d like to succeed in this journey.”*

Response from Executive 1: *“I think it’s a shared vision inside the company because for example we have a kind of a team based meetings. In these team base meetings, we have discussion about it, for example, investments, in case of having a kind of investments or buy new machines, it’s not the decision of our CEO, no, it’s the decision of the team.”*

Response from Executive 2: *“Actually in <company> we do a process and we made the vision together, and all of us shared the dream for <company>, how we see <company> in 20 years, and how we’ll achieve this dream.”*

Case #5:

Response from the CEO: *“We even have a story to explain each one of those [core values], so that everyone will really understand what they mean and has an example and we are using in our hiring and firing processes.”*

Response from Executive 1: *“We share [the core values] with them and it’s been more seeing it in action, every time we do something it’s not just the words we say as to what we do and that’s really the only way I think we can really show them that we are not just saying and we are doing it and it comes from leadership, it goes all the way down from the supervisors down to the crew. It’s just the culture.”*

Response from Executive 2: *“It’s a culture thing you know. It’s all about how you approach situations. It’s the little things and it’s the little help… right on the frontline with you making sure you are good. I find that if you can really share the vision of what we are doing and helping people, then they are on board. They’re willing to sniff out the problems too --- hey, if we do this way, it might be better. You know when you get that buy-in, it’s almost like if they were an owner.”*

### 3.2 Finding #2—higher compassion tended to be expressed along with a long-term business perspective

Five of the seven case companies showed convergence on the theme of compassion in their organizations as a component of quality relationships. Two of the TECs shared powerful stories and expressions of compassion among those five. They also showed a consistent willingness to de-emphasize short-term profits in favor of acting in a way that was consistent with their values, demonstrating a firm belief in first treating people well.

The CEO of Case Study #6 expressed this concept best:

*“What would be best for our company long-term? The way we approach business and making decisions may not be good for short-term profit, but we believe, will have tremendous benefits to the community, to our people, to our customers… and it may be a five, ten-year type or generational type of investment.*

*But if we wanted to be purely profit driven, we would absolutely go and make dramatic cuts to our payroll, we would reduce our inventory investment…we would pull out probably 80% of our community involvement…*

*So, there are absolutely a lot of those different decisions that if we really wanted to enhance and kind of compare ourselves to the big boxes and things like that, we could absolutely get there. **We already know the path; it’s something though we refuse to do.**”*

From a human behavioral perspective, there is evidence that an increase in compassion would naturally correlate with a lower focus on task-oriented activities that would lead to maximizing short-term profit. Jack et al. have demonstrated through brain imaging that empathetic concern, and spiritual faith, are negatively correlated with analytic thinking. They report that “there is mounting evidence, both correlational and causal, which demonstrates that analytic thinking (as measured by tests of intelligence and critical thinking) discourages the acceptance of religious and spiritual beliefs… a number of studies and theories have linked measures of empathy, social cognition, and emotion self-regulation—including measures of social and emotional intelligence—to religious and spiritual belief” (Jack et al., 2016).

### 3.3 Finding #3—positive core values were expressed more prominently than explicit greater mission/purpose

Only one of the seven cases demonstrated convergence on an explicit greater purpose or mission for the business. For the other six, it was expressed as a set of core values that would drive and inform all decisions and actions. Only one PIC showed convergence on an explicit greater purpose (Case #2). The other three PICs showed strong convergence toward a set of core values. From the Chinese Eastern perspective, it was described as a direction.

My expectation prior to the interviews was that the PICs would all be driven by an explicit greater purpose, while the TECs would express their mission/purpose in terms of serving customers or some other conventional business measure. The prevalence of core values rather than explicit purpose suggests, perhaps, that the leaders in these organizations, whether PIC or TEC, are placing a greater emphasis on ‘who they are being’ rather than on ‘what they are doing’ through their organizations.

The following excerpts illustrate responses that expressed positive core values rather than an explicit greater purpose:

Case #4, Executive 2: *“Six core values… we have mindfulness, stewardship, value add, prudence, strategic mindset, and collaboration… this has been distilled into these six values and integrated in our HR processes.”*

Case #5, Executive 1: *“I think that wraps it all is we love helping people and that was like the core at all… core values and it was where family loves helping people.”*

Case #5, CEO: *“The mission statement of yesteryear today, I do not think has a place in business. I think it should be translated into core values and they should be very simple and they should be absolutely used in every single decision that the business makes both strategically and non-strategically. They are very simple, ours is three. Number one, we are a family that loves helping people. Number two, our integrity ensures the best customer experience, and number three, our world class vision and continuous improvements honors us to be the very best.”*

Case #6, Executive 1: *“… you can see around the room we have our [10] core values here. [developed] through an exhaustive process.”*

Case #6, Executive 2: *“We have the culture, we have our core values, it’s funny to me is I manage by those core values because they are so important. Did you see the board that we have on the wall? It’s a core values in action board. Basically we have all of the ten core values in cards, … and we encourage this staff, this is them praising each other, thanking each other for helping with this, helping with that…*”

### 3.4 Finding #4—the primary difference between PICs and TECs is related to mindset of connectedness and reflective practices

All four of the cases identified as PICs showed convergent presence of mindset of connectedness and reflective practices. None of the three TECs showed convergence on the presence of mindset of connectedness, and only one showed convergence on reflective practices. This finding represents the primary differentiator between the PICs and TECs.

It also aligns with the literature suggesting that a relationship exists between reflective practices and the development of a mindset of connectedness, which in turn manifests in the effort to create positive impact outcomes through the organization.

The following excerpts illustrate responses from PICs relative to reflective practices and mindset of connectedness:

Case #1, CEO: *“When we work our individual time or when we work our group time around connectedness, we [use] a little Tibetan prayer bowl and take a few minutes to breathe and connect with ourselves not the most unique thing. We do a personal check in, where people express, you know what’s running through them at the time.”*

Case #1, Executive 1: *“I’m an avid hiker, I am outside all the time, I meditate daily, I find -- I go back and forth in that practice but I feel that I’m way stronger when I’m meditating, I do yoga, I’m an active reader…”*

Case #1, Executive 2: *“Personal: I committed myself to one hour of meditation a day and every day, and I get up at 4:30 and sit on my cushion… Organizational: We [have] the big breath break. …where we are teaching people with modern psychology we call stress reduction, mindfulness therapy, you know, meditation and following the breath.”*

Case #2, CEO: *“I think the practices of <company> in particular, like the daily stand-up meeting, where we all gather in a circle and share whatever is on our minds that are important.”*

Case #3, CEO: *“When we started those summits, we started on small teams. So, we started with 12 and then we got to 25 person and then we got to 45 and then we have done -- with one company, we got the 250 and then last year, this year we got the 400 together… So, we say whenever you are in doubt just enlarge the circle.”*

Case #3, Executive 2: *“I regularly pray in church. From time to time I go to three days retreat outside.”*

Case #4, Executive 1: *“Meditation today has become my prayer time. So that’s very important to me. So my quiet time in the morning is my meditation. And now, mindfulness has become a very, very big thing in my life, and I’ve actually kind of like made it part of my practice.”*

Case #4, Executive 2: *“I do prayer practice. And I practice a lot about being, I would say, being grateful to whatever I have. And over time I also do quite a bit of mindfulness practices. And specially when I’m overwhelmed I would just take a few deep breaths that help me.”*

### 3.5 Finding #5—the primary activity relative to environmental outcomes remains at the level of doing less harm

Three of the four PIC companies demonstrated convergent presence relative to the construct of social/environmental outcomes. This was primarily driven by activities related to human interaction and partnering with companies that shared their core values. With regard to the environmental aspect, all of the companies (whether PIC or TEC) reported approaching environmental impact from a ‘do less harm’ perspective rather than a ‘regenerative’ perspective. Indeed, this may be the most challenging transformation for many of the companies identified as PICs.

While extensive research has explored the positive business case for corporate social responsibility (Nidumolu et al., 2009; Laszlo and Zhexembayeva, 2011; Eccles et al., 2014), most companies remain focused on doing less harm rather than creating good relative to environmental impact. For example, many CSR initiatives aim to use less energy and less water and to produce less waste, yet studies by Ehrenfeld and Hoffman (2013) and Steffen et al. (2013) suggest that the resulting collective economic activity continues to worsen environmental and social indicators of health and wellbeing, whether measured in terms of climate change, biodiversity loss, nitrogen cycle disruption, employee disengagement, social exclusion, or personal wellbeing.

While regenerative environmental impact is part of the ideal of many forward positive institutions whose aims are flourishing, prosperity, increased wellbeing, and resilience (Seligman, 2011; Laszlo et al., 2014), the findings of this study suggest that this is a conceptually challenging idea to put into practice, even for those organizations committed to positive impact.

## 4 Discussion

The focus of this study was aimed at two leverage points within the model depicted in Figure 1: first, how the quality of relationships in an organization affects the ability to translate the leader’s mindset, practices and purpose into positive impact for all stakeholders; and second, how organizations that set out to achieve positive societal impact (PICs) differ, if at all, from traditional successful business organizations (TECs).

The literature offers extensive exploration of individual motivation and behavior in organizations, of dyad or team-level interactions, and of organization-wide behavior. An opportunity exists within the literature to explore the thread that connects the layers of activity from individual leader to the organizational outcomes, specifically in the context of positive societal impact. This is what animates the comparison of the seven case studies in this analysis, with the goal of gaining insights about the influence of quality relationships within the dynamic of generating positive impact.

The selection of the case companies was intended to provide a level of similarity in terms of company size and ownership to facilitate comparison, while providing points of differentiation in terms of industry, nationality, and intent to generate positive impact as PICs. The findings suggest that the emergence of positive impact is not related to any particular industry. The cases include industries such as manufacturing and shipping, which are stereotypically not associated with PIC-like organizations. Yet each of the seven case companies can be considered a positive corporate citizen. The effort to create positive outcomes through business is also not related to a particular country or region. Whether based in the US, Egypt, or Singapore, while the cultural expression was varied, the underlying quality of relationships and concern for stakeholders was consistent.

The findings of the case comparisons and individual interviews offer insights into both of the main areas of focus. Findings #1 and #2 relate closely to quality relationships, with shared vision and compassion as two central elements of quality relationships defined by the literature. Findings #3 and #4 relate to the differences between PICs and TECs, and the theoretical themes that determine the difference between the two types of organizations. Finding #5 illustrates the considerable distance still to be traveled for business organizations relative to environmental outcomes and moving beyond ‘reducing harm’ toward generating positive environmental impact.

Finding #1 offers the strongest evidence—across all seven case companies—of the critical role that shared vision plays in the translation of intention into outcomes as part of quality relationships. At one level, this finding serves as an affirmation of the theoretical construct of relational climate as described in the literature. Taken further, five of the seven case companies demonstrated convergence on all three elements of relational climate. One of the PICs, Case #2, did not show convergence on the measure of compassion while one of the TECs, Case #7, did note show convergence on relational energy or compassion.

Finding #2 relates to compassion as a component of quality relationships. It was found as a convergent trait in five of the seven case companies. One PIC and one TEC each did not demonstrate convergence for compassion. The conversations related to compassion were primarily about treating people well in the organization, caring about employees, creating an environment of flourishing and wellbeing. There was also a perspective on business economic performance that was long-term oriented, and an unwillingness to treat people poorly to achieve a momentary financial gain.

The intersection of several of the findings provides an additional perspective. For example, all seven organizations demonstrated elements of quality relationships regardless of *a priori* categorization. In Finding #1, whether PIC or TEC, the interviews demonstrated a strong presence of shared vision across all seven cases. In Finding #3, the interviews showed convergence around strong core values or greater purpose in six of the seven cases. Taken together, the intersection of shared vision and strong core values offers a strong potential explanation of the high quality relationships observed in the case companies. The combination of positive core values and shared vision is a powerful one.

In a further illustration of the intersection of several themes in the findings, the five cases that showed convergence on core values were the same five cases that showed convergence on all three components of quality relationships. In other words, there was a 100% match between core values and quality relationships. In the cases where core values were not convergent, elements of quality relationships were also missing.

Both of these illustrations of intersection between the findings provide an avenue for considering the thread between layers within the theoretical model in Figure 1. The second area of focus for this study was to explore the comparison points between PICs and TECs. The expression of core values in Finding #3 alone does not appear to move an organization to the full PIC category.

The primary differentiator between the PICs and the TECs appears to reside in a mindset of connectedness combined with the use of reflective practices by leaders. This affirms several studies cited earlier, suggesting a strong relationship between reflective practices, a mindset of connectedness, and a perception of business as serving a greater good. It also suggests that the intention to achieve positive impact is itself an important factor, and that reflective practices by leaders are indeed influential in creating a mindset of connectedness that can be transferred through quality relationships in the organization.

All four PICs showed convergent presence of both a mindset of connectedness and the use of reflective practices. None of the TECs showed convergent presence on both of these dimensions together, with only one TEC, Case #7, showing presence of reflective practices. This intersection of findings provides the clearest answer to the question of how organizations that set out to achieve positive societal impact differ from traditional successful business organizations. There is a shift in mindset among leaders toward one of connectedness, and it is driven by the use of reflective practices.

In previous studies of positive impact leaders from the literature, for example Tsao and Laszlo (2019), the analysis was based entirely on the perceptions of individual leaders speaking for themselves and their companies. A contribution of this study, therefore, is to integrate the perceptions of several individuals from the same organization, including the CEO, and align those perceptions to assess the extent to which the intent toward positive impact outcomes is shared. The findings suggest that for PICs the alignment exists at the level of the organization as well as at the level of the individual leader. The leaders of PICs tend to engage in reflective practices and develop a mindset of connectedness that radiates into their organizations.

I initially categorized the seven case companies as PICs or TECs based on whether they were active in organizations dedicated to a positive impact mission. The theoretical framework offered nine measuring points on which to determine the extent to which each case company demonstrated positive impact, based on whether the individual leaders from the organization expressed convergent views in separate interviews.

An overall evaluation of the seven cases indicates that two of the PICs showed convergence on eight of the nine measures, one PIC showed convergence on seven measures, one PIC on six measures, and all three TECs on four measures (see Table 1). From this evaluation perspective, there is a clear separation between the PICs and TECs, with the PICs indeed aligning more closely along the theoretical components of positive impact organizations.

### 4.1 Limitations

While the cases offer an opportunity for a rich level of analysis, several limitations remain. The case study companies were of similar size range, all privately held. However, they were not from the exact same industry. The selection of interview subjects inside the company was dependent on introductions from the CEO or the CEO’s office indicating the potential for selection bias. However, in five of the seven cases, my request for the head of HR and a VP of Operations was possible. These roles exist and are staffed by the current occupant of the position, regardless of whether their perceptions of the topic of the study align with the referring CEO.

The findings could have been affected by the selection criteria. A further study based on an assessment of independent performance data, rather than affiliation with any given groups, would allow a further replication and clarification of the findings.

The potential for single analytical perspective exists. The coding, analysis and interpretation of the case study interviews was done by myself as a single investigator. While I followed a structured and consistent methodology in the observation, labeling and interpretation of the data, my interpretations nonetheless reflect the perspective of a single viewpoint, and as such has the potential for bias. A next step in this process might be to conduct a multi-rater analysis to gain multiple perspective on the case interpretation, while assuring inter-rater reliability using methodologies such as those described by Boyatzis (1998, pp. 152–159).

### 4.2 Conclusion

The people from the three TECs that I interviewed were inspiring and forward-thinking business leaders. Their presence and approach affirm findings from other studies that a shift in business thinking is occurring more widely than previously thought. The people from the four PICs were likewise positive and engaging. Through all of the interviews for this study, the interview subjects displayed obvious passion for their companies, and energetic willingness to discuss their experiences.

The findings of this research suggest that the evolution of thinking about the role of business in society, and the shift in mindset and consciousness in business leaders toward one of positive impact, is messy, nonlinear and complex. There is a spectrum of positive impact that does not emerge neatly.

If this is the middle of a normal distribution, and the TECs interviewed for this research represent the mean of current business practice, then it is cause for great encouragement. All three TECs are outstanding companies, with positive energy, caring interactions and collaboration, strong positive cultures, and a perspective focused on creating long-term shared value. My sense is that these companies do not represent the mean, however. They are closer to the PIC end of the scale than the average company today, yet they represent the trend toward business as a force for positive impact in society.

One area for further study might be to address the difference between privately held companies versus those that are publicly traded, and to explore the extent to which publicly traded companies have the same freedom to pursue positive impact. All seven cases in this study were privately held, which was an intentional design choice to facilitate comparison.

In summary, successful TECs and PICs exhibit many commonalities of positive impact principles, such as positive core values and an emphasis on human interaction and quality relationships. Shared prosperity tends to be seen in terms of long-term financial performance. Whether PIC or TEC, positive impact leaders exhibit common elements such as the centrality of positive core values; emphasis on collaboration and caring in their organizations; and delivering positive outcomes for all stakeholders. The addition of reflective practices and a mindset of connectedness appears to be the differentiator that shifts a company fully into the PIC column, and it offers an area for further study and exploration.

## Data availability statement

The raw data supporting the conclusions of this article will be made available by the authors, without undue reservation.

## Ethics statement

The studies involving humans were approved by Case Western Reserve University, Weatherhead School of Management, Institutional Review Board. The studies were conducted in accordance with the local legislation and institutional requirements. The participants provided their written informed consent to participate in this study.

## Author contributions

The author confirms being the sole contributor of this work and has approved it for publication.
